# New Biodegradable Carboxymethyl Cellulose-Based Films with Liquid Products of Wood Pine Pyrolysis with Antibacterial and Antioxidant Properties

**DOI:** 10.3390/ma18102228

**Published:** 2025-05-12

**Authors:** Grażyna B. Dąbrowska, Marcel Antoszewski, Aleksandra Szydłowska-Czerniak, Aneta Raszkowska-Kaczor, Tomasz Jędrzejewski, Sylwia Wrotek, Monika Bartkowiak, Maria Swiontek Brzezinska, Magdalena Zborowska

**Affiliations:** 1 Department of Genetics, Faculty of Biological and Veterinary Sciences, Nicolaus Copernicus University in Toruń, Lwowska 1, 87-100 Toruń, Poland; mant@doktorant.umk.pl; 2 Department of Analytical Chemistry and Spectroscopy, Faculty of Chemistry, Nicolaus Copernicus University in Toruń, Gagarina 7, 87-100 Toruń, Poland; 3 Łukasiewicz Research Network–Institute of Polymer Materials and Dyes Engineering, M. Skłodowskiej-Curie 55, 87-100 Toruń, Poland; 4 Department of Immunology, Faculty of Biological and Veterinary Sciences, Nicolaus Copernicus University in Toruń, Lwowska 1, 87-100 Toruń, Poland; 5 Department of Chemical Wood Technology, Faculty of Forestry and Wood Technology, University of Life Sciences, Wojska Polskiego 38/42, 60-637 Poznań, Poland; monika.bartkowiak@up.poznan.pl (M.B.);; 6 Department of Environmental Microbiology and Biotechnology, Faculty of Biological and Veterinary Sciences, Nicolaus Copernicus University in Toruń, Lwowska 1, 87-100 Toruń, Poland

**Keywords:** antibacterial film, antioxidant properties, bioactive material, methylcellulose, pine tar, wood pyrolysis products

## Abstract

Novel carboxymethylcellulose (CMC) films with liquid products of pyrolysis (LPP) from wood pine were produced. The obtained CMC-LPP films were plasticized with 5% glycerol. CMC-LPP films were a light brown colour with a characteristic smoky scent, and showed a higher oxygen permeability when compared to control film without the addition of the LPP. CMC-LPP exhibited high antioxidant activity (5 and 18 times higher than CMC films). Furthermore, the antibacterial activity of the CMC-LPP films was tested, showing a strong inhibiting growth effect on the seven tested human pathogenic bacteria. The new material had the most substantial bacteriostatic effect on *Listeria monocytogenes*, *Salmonella typhimurium*, and *Pseudomonas aeruginosa*. Introduction of LPP to plasticised CMC produces an eco-friendly material with biocidal effect and favourable mechanical and structural properties, which shows its potential for possible use in many industries.

## 1. Introduction

Synthetic polymers constitute a severe threat to the environment since most polymers used are not biodegradable, leading to their accumulation in soil and water [1]. There is an urgent need to develop technology for the biodegradation of synthetic polymers most widely used in food packaging, i.e., low-density and high-density polyethylene, polyethylene terephthalate, polylactide, polypropylene, polyvinyl chloride, and polystyrene [2,3]. One of the solutions that could hinder plastic pollution might be the development of packaging from renewable sources or materials with the addition of natural products that would increase the biodegradability of produced polymers [4,5]. One of the possibilities is the use of cellulose derivatives in the packaging industry or agriculture. Cellulose is the main structural component of cell walls in lower and higher plants and cellulose-rich materials include trees, cotton, flax, hemp, jute, grasses, and agricultural residues [6]. Cellulose polymer is mainly used to produce paper, cardboard, and cellophane. Cellulose forms non-water-soluble fibres, which are sturdy and flexible. It is often necessary to process cellulose into solubilized forms for use in the industry [7].

One of the fastest-growing economy sectors is the packaging sector, with an expected growth of the packaging industry of over 3% by 2028, reaching approximately USD 1.2 trillion [8]. Moreover, with new regulations aiming at reducing plastic pollution, emerging areas of the packaging industry are eco-friendly packaging, smart or active packaging, and flexible packaging with antioxidant, antiviral, and antibacterial properties being of special interest [8,9]. Polysaccharides have gained exceptional interest as potential replacements for conventional plastic packaging due to their edibility, good barrier properties, and antibacterial properties, enabling a longer shelf-life of packaged goods with the use of polysaccharides-derived packaging (e.g., pectin, carboxymethylcellulose, chitosan) [10,11]. Carboxymethylcellulose (CMC) is an anionic, water-soluble cellulose derivative, a linear polysaccharide of anhydroglucose. β-1,4-glycosidic bonds connect the polymer units of CMC; some anionic carboxymethyl groups (i.e., –CH_2_COOH) in the structure of this polymer replace hydrogen atoms from some hydroxyl groups present in unmodified cellulose [12]. The application of CMC highly depends upon the purity, degree of polymerization, degree of substitution, and uniformity, which impact the properties of the resulting products (i.e., solubility, particle size, rheological properties, viscosity, and others) [13]. CMC is generally recognized as safe (GRAS) and is used as a stabilizer or thickener in various foods [14]. CMC-based hybrid materials have a wide range of applications in the biomedical, pharmaceutical, textile, construction, food, plastics, cosmetics, paper, and oil industries. Due to its biodegradability and high availability, CMC shows excellent potential as a new component of eco-friendly materials [15,16,17]. However, challenges such as poor water resistance, mechanical fragility, and limited scalability remain significant for large-scale production and application. To address the above-mentioned limitations, current research areas focus on the chemical modification of CMC, composite formulations, and incorporation of bioactive agents that improve performance and functionality [10,11].

Due to the possibility of undesirable contact between microorganisms and food products, it is necessary to limit and combat the spread of human pathogens [18,19,20,21]. The World Health Organization (WHO) estimates that around 600 mln cases are associated with foodborne diseases, including 420,000 deaths yearly [22]. Primary foodborne pathogens include *Escherichia coli*, *Bacillus* spp., *Listeria monocytogenes*, *Salmonella* spp., and *Staphylococcus aureus* [23]. Other sources of infections are hospitals, i.e., hospital-acquired infections or healthcare-associated infections (HAIs) [24]. These infections usually manifest around 48–72 h after admission. They are primarily associated with central line (i.e., bloodstream infections), catheter (i.e., urinary tract infections), surgical site (i.e., infections acquired in the operating room), and others [24,25]. The most common causes of HAIs are *Clostridium difficile*, *S. aureus*, *Enterococcus* spp., *Pseudomonas aeruginosa*., *Klebsiella* spp., *Staphylococcus* spp., and *Candida* spp. [26]. One of the promising solutions to limit the spread of foodborne pathogens and HAIs is antibacterial packaging, i.e., active packaging that contains antimicrobial agents. This approach allows us to push the boundaries of food safety, preservation technology, and prevention of infections, including HAIs [27]. There are many reports about antimicrobial and antioxidant films with the addition of bioactive components such as essential oils [28], bergamot, lemongrass, rosemary oils [29], tea tree oil [30], phenolic acids [31], and others. Recently, there has been a spike in research on materials based on the CMC matrix with different additives like amino phenylpropanoic acid, a biological and anti-cancer active compound [32], chitin, and spores of *Trichoderma viride* with antifungal properties for use in agriculture [33].

An exciting product of plant origin is tar obtained by dry pyrolysis of wood or bark of deciduous and coniferous trees (e.g., birch, pine, cedar, and juniper). Tar contains phenolic compounds, benzene, xylene, phytoncides, organic acids, ketones, aliphatic alcohols, resin substances, and tar substances [34]. Wood pyrolysis products have bactericidal, antiseptic, and anti-inflammatory properties and are used in medicine, veterinary, and agriculture [35,36,37]. Tar contains volatile substances with antimicrobial properties; therefore, materials with pyrolysis products incorporated into the polymer matrix are promising products for use in the packaging industry. Packaging with antimicrobial activity has great potential and can largely contribute to reducing food waste problems. Furthermore, adding natural substances to the polymers may increase the biodegradability of produced polymers, reducing the input of synthetic polymers polluting the environment [38,39]. On the other hand, liquid pyrolysis products (LPP) are a rich source of antioxidants, mainly phenolic compounds, whose content depends on wood types, pyrolysis fractions, and operation conditions, such as temperature, heating rate, and residence time [40,41]. Phenolic compounds with potent antioxidant and free radical scavenging activities in LPP are pyrolytic products of lignin and hemicellulose and comprise 30–60% of the total organic compounds [42]. Among them are phenolic acids, phenols, polyphenols, flavonoids, phenoxy species, benzene derivatives, and others [41,43]. Therefore, biodegradable polymer packaging materials fortified with LPP have potential to prolong the shelf life of food products by delivering natural preservation components in a controlled manner. Moreover, the packaging films loaded with LPP containing phenolic derivatives, especially syringol and guaiacol, may affect the sensory properties of food products, giving them smoked, roasted, and grilled aromas and flavour [44].

This study aimed to produce and characterize carboxymethyl cellulose-based bioactive materials with antibacterial and antioxidant properties. We used liquid products of pine wood pyrolysis (LPP) without tar as an additive. We assumed that adding LPP to the membrane made on the CMC matrix, known for its antibacterial properties, would result in a membrane exhibiting antimicrobial and antioxidant properties. We formulated the following questions: (i) How does LPP incorporation affect CMC films’ antioxidant and antibacterial properties? (ii) How does LPP affect the mechanical and cytotoxicity profiles of the produced films? (iii) How does the addition of LPP influence the biodegradability of the composite films? We aimed to develop a multifunctional, eco-friendly packaging material by addressing these questions.

## 2. Materials and Methods

### 2.1. Products of Pyrolysis

Liquid pyrolysis products were obtained from approximately 2000 g of chipped wood pine wood via the pyrolysis process in laboratory conditions, as detailed in the article [45]. Thermal decomposition of pine wood was carried out with limited oxygen access to the assumed final temperature at 550 °C, with a heating speed of approximately 3 °C/min. Steam gases produced as a thermal decomposition product were directed from the retort to the cooling system, where some condensed to form a liquid product. The liquid pyrolysis product used in this study was tar-free. The composition of LPP used in this study was previously characterized by Bartkowiak et al. [34,45] and contains a diverse range of bioactive compounds, including phenolic acids (e.g., gallic acid, protocatechuic acid), phenolic aldehydes (e.g., 3,4-dihydroxybenzaldehyde, 4-hydroxybenzaldehyde), flavonoids (e.g., catechin, apigenin), and other polyphenols, along with volatile organic compounds (VOCs) such as aliphatic alcohols, ketones, and benzene derivatives.

### 2.2. Film Preparation

CMC (5% *w*/*v*; Sigma-Aldrich, Poznań, Poland) was mixed in distilled water heated to a temperature of 80 °C using a heating magnetic stirrer (VELP MH 15, Velp Scientifica, Düsseldorf, Baden-Württemberg, Germany) at 500 rpm for 3 h. Glycerol (5% *v*/*v*) was mixed with CMC solution and stirred for 20 min at a temperature of 60 °C. The liquid pyrolysis product (LPP) was added into the CMC solution with continuous stirring for 30 min to 5% (*v*/*v*) final concentration. New films on the CMC matrix with liquid pyrolysis products were obtained and marked CMC-LPP. As a control, CMC films without the addition of LPP were also produced. Solution (35 mL) was poured on plastic plates sized 110 mm × 110 mm and kept under the fume hood for 72 h at 25 °C. The thickness of CMC-LPP films was investigated by measuring the thickness at ten different points using a digital micrometer (Fanger, Kraków, Poland). Results were presented as average values with standard deviation (SD).

### 2.3. SEM Analysis

The morphology of the samples was studied using a scanning electron microscope (SEM) Hitachi SU8010 (Tokyo, Japan, 2011). The microscope has a cold cathode with field emission, two SE detectors, a BSE detector, and an EDX detector for X-ray microanalysis. The CMC and CMC-LPP films were covered with gold before the observation using a Cressington Sputter Coater with a module for measuring the thickness of the sputtered gold layer (Cressington Scientific Instruments, Watford, UK).

### 2.4. Uniaxial Tensile Tests

Uniaxial tensile tests were performed with an EZ-Test SX Texture Analyzer (Shimadzu, Kyoto, Japan). Samples were paddle-shaped and stretched until rupture with a stretching speed of 30 mm/min. Trapezium X software version 1.4.5 (Shimadzu, Kyoto, Japan) was used to calculate Young’s modulus (E), the maximum stress that the sample can withstand (σ_max_), and strain at break (ε).

### 2.5. Oxygen Permeability

Oxygen permeability was tested according to ASTMF 1927 [46], the standard test method for the determination of oxygen gas transmission rate, permeability, and permeance at controlled relative humidity through barrier materials using a coulometric detector. The analyses were performed using MultiPerm O_2_-CO_2_ DC (PermTech, London, UK, B02Y0). Both conditioning and measurements of films were performed at 23 °C and 0% humidity. Five repetitions were performed for each of the three tested samples. The tests were conducted using the following parameters: carrier flow—12.06 mL/min; automatic barometric pressure compensation was applied.

The measurement was carried out in two phases. During the first phase (conditioning), anhydrous nitrogen was passed through the chambers to purge both the upper and lower measurement chambers of residual gases. This stage lasted for 8 h for each sample. The second phase was the actual measurement, during which oxygen flowed. The measurement needed to achieve the saturation curve ranged from 1 to 1.5 h, depending on the sample.

### 2.6. Evaluation of the Antioxidant Properties of Films

In the present study, the modified QUick, Easy, New, CHEap and Reproducible (QUENCHER)-2,2-diphenyl-1-picrylhydrazyl (DPPH) and QUENCHER cupric ion reducing antioxidant capacity (CUPRAC) procedures previously described in Tymczewska et al. [47] were applied for the evaluation of the antioxidant properties of two solid film samples: CMC-LPP and CMC (control). The AC was determined in three replications, and the results were expressed as μmol Trolox equivalents (TE) per g of film sample.

For the DPPH test, each film sample (0.1 g) was ground in an electric laboratory mill (FW100, Chemland, Stargard Szczeciński, Poland), and 6 mL of DPPH solution (304.0 μmol/L) was added to a test tube containing the sample. Then, samples were shaken vigorously (Classic Vortex Mixer, Velp Scientifica Srl, Usmate, MB, Italy) for 10 min to facilitate the reaction with the reagent. After shaking, test tubes were put in the dark for 15 min. After incubation, the absorbance of the optically clear supernatant was measured spectrophotometrically at 517 nm using a Hitachi U-2900 spectrophotometer (Tokyo, Japan). For the CUPRAC method, 0.1 g of film sample was added to 10 mL of mixture solution (2 mL of copper chloride, 2 mL of ammonium acetate buffer, 2 mL of neocuproine, 3 mL ethanol, and 1 mL redistilled water) and shaken vigorously for 10 min to facilitate the reaction with the reagent. After 20 min of incubation at room temperature in the dark, the absorbance of the optically clear supernatant was measured spectrophotometrically at 450 nm.

### 2.7. Assessment of the Antibacterial Properties of the Film

Bacterial strains used in this study come from the collection of bacterial cultures of the Department of Genetics at the Nicolaus Copernicus University in Toruń: Escherichia coli DH5α, *Salmonella typhimurium* T98; the American Type Culture Collection (ATCC): *Staphylococcus aureus* ATCC 6538P; and the Polish Collection of Microorganisms (PCM): *Enterococcus faecium* PCM 2787, *Campylobacter jejuni* PCM 2852, *Listeria monocytogenes* PCM2606, and *Pseudomonas aeruginosa* PCM 3035. Bacterial strains were stored at −80 °C in glycerol stocks.

LB medium (NaCl 10 g, casein peptone 10 g, yeast extract 5 g, 15 g agar, dH_2_O 1 L) was inoculated with a liquid (LB medium) bacterial culture at a 1.5 × 10^8^ colony-forming unit per millilitre concentration. CMC or CMC-LPP film of 20 mm × 20 mm was placed in the middle of the plates. Three iterations were made for each bacterial strain. Petri dishes were incubated for 20 h at 37 °C. After overnight incubation, the zone of inhibition was measured.

### 2.8. Cytotoxicity Analysis of Film

The L929 fibroblast cell line (NCTC clone 929) of murine origin was obtained from the American Type Culture Collection (ATCC, Manassas, VA, USA). Cells were maintained in RPMI-1640 medium supplemented with 10% heat-inactivated fetal bovine serum (FBS), streptomycin (100 µg/mL), and penicillin (100 IU/mL) at 37 °C under 5% CO_2_. All cell culture reagents were sourced from VWR International (Radnor, PA, USA).

For cytotoxicity assessment, CMC and CMC-LPP films were cut into 6 cm × 6 cm sections and sterilized by UV light exposure for 30 min on each side. The surface-area-to-extraction-medium ratio was maintained at 6 cm^2^/mL. Samples were incubated in RPMI-1640 medium at 37 °C with 5% CO_2_ for 8 h to obtain extracts. Preparation of the samples followed the ISO 10993-12 standard [48]. Control media, not exposed to any material, were subjected to identical conditions and served as negative controls. After extraction, supernatants were centrifuged at 2000× *g* for 15 min, and the resulting media were stored at 4 °C for no longer than 24 h prior to application.

L929 cells were seeded onto 96-well plates at 5 × 10^3^ cells/well density and pre-incubated for 24 h. For 24 and 48 h treatments, extracts were prepared by diluting them in RPMI-1640 medium at ratios of 1:10, 1:3, and 1:2, as well as being undiluted (100% extraction medium). Control groups were maintained in corresponding dilutions of untreated culture medium.

Following stimulation, cell metabolic activity was assessed using the MTT assay (Merck KGaA, Darmstadt, Germany). Post-treatment, cells were washed once with PBS, and 100 µL of MTT solution (0.5 mg/mL) was added to each well. Plates were incubated at 37 °C for 3 h. Subsequently, 50 µL of DMSO was added per well and agitated horizontally for 10 min using a microplate shaker. Absorbance was measured at 570 nm, with background correction at 630 nm, using a Synergy HT plate reader (BioTek Instruments, Winooski, VT, USA). Cell viability was calculated as a percentage relative to control cells cultured in an equivalent dilution of untreated medium (set at 100%). Blank controls included wells containing only the extracts or medium without cells. The MTT assay was conducted independently in three biological replicates.

### 2.9. Biodegradation of CMC and CMC-LPP Films

Biodegradability of the produced films in the soil was assessed with the OxiTop system (WTW, Wrocław, Poland). In total, 100 g of soil was placed in OxiTop jars and mixed with fragments of CMC or CMC-LPP films (total mass 1 g, 2.0 cm × 2.0 cm) with or without the addition of fungal spore suspension of *Trichoderma viride* at a concentration of 1.5 × 10^6^; control jars contained soil without the film. Jars were incubated for 21 days at 26 °C. The assessment of the biodegradability was based on biochemical oxygen demand (BOD) and expressed as mgO_2_/kg of soil. Organic carbon (301 g/kg) and nitrogen (21 g/kg) content in the soil was measured via Tiurina and Kjeldahl methods, respectively. The content of other tested compounds in the soil was as follows: P_2_O_5_ (24.2 mg/100 g), K_2_O (22.5 mg/100 g), Mg (6.3 mg/100 g), N-NO_3_ (25 mg/kg), and NNH_4_ (2.32 mg/kg) measured according to PN-ISO 10390:1997 [49], PN-R-04022:1996 [50], PN-R-04022:1996/Az1:2002 [51], PN-R-04020:1994/Az1:2004 [52], and PN-R-04028:1997 [53]. Abundance of native microbes in the soil was tested beforehand via the Koch plate method, with results presented in Table 1.

### 2.10. Statistical Analysis

The results of antibacterial and antioxidant assays were analyzed with the Past 4.12b programme [54] using a one-way analysis of variance with the post hoc Tukey test. The level of significance was set at *p* < 0.05.

Cytotoxicity data were evaluated using GraphPad Prism 7.0 (GraphPad Software Inc., La Jolla, CA, USA). Statistical analysis was performed with one-way ANOVA followed by Tukey’s post hoc test. Data are expressed as mean values ± standard error of the mean (SEM). A *p*-value of less than 0.05 was considered statistically significant.

## 3. Results and Discussion

### 3.1. Obtained Films

**Figure 1:**
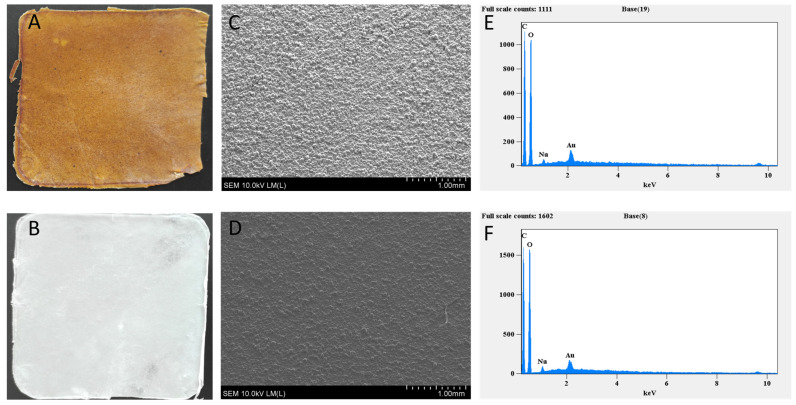
Photographs (**A**,**B**), scanning electron microscopy (SEM) images (**C**,**D**), and X-ray microanalysis (**E**,**F**) of the surfaces of CMC-LPP (top row: (**A**,**C**,**E**)) and control CMC films (bottom row: (**B**,**D**,**F**)).

The CMC-LPP membranes had a thickness of 0.074–0.081 cm with a light brown colour and a characteristic scent coming from volatile compounds contained in pine tar (Figure 1).

It is well known that packaging colour plays a crucial role in consumer visual perception and their purchasing decisions. Therefore, the darkening of film and reduction in light transmission are unacceptable for consumers who prefer transparent packaging. However, darker packaging materials offer advantages for food products susceptible to oxidation processes and colour changes, contributing to their preservation by delaying spoilage, lipid and protein oxidation reactions, and nutritional loss.

Similarly, the CMC films containing polyphenol-rich extracts from coffee husk and carbon dots prepared using the biowaste residue of coffee husk extraction displayed a brownish hue due to the inherent brown tint of these additives. Nevertheless, the transparency of the fortified films was sufficient and acceptable because the covered green plant remained visible [55]. Moreover, CMC films loaded with polysaccharides from mulberry leaves, apple skin extract, lignin samples extracted from grapevine using deep eutectic solvents, and NaOH became darker, greener, and yellower, which can be attributed to the presence of polyphenol compounds, including flavonoids and anthocyanins in the added active agents [14,56,57].

On the other hand, the volatile compounds, including terpenes with characteristic odour, present in LPP, added to the CMC film, can be effective as flavouring agents and mask the intense aroma of the packed food products. In contrast, high concentrations of added active components with intense aromas to film material can lead to high release rates, which limit their direct application for long-term food packaging. Therefore, further research is needed to optimize the application of aromatic active additives to biopolymers and address the challenges related to their pungent odour and stability in various food systems.

It can be noted that the addition of LPP to CMC film caused a significant decrease in all evaluated mechanical parameters, such as Young’s modulus (E), stress (σ), and strain (ε) at break (Table 2). The formation of intermolecular interaction between hydroxyl groups of CMC and LPP reduced the mechanical parameters of CMC-based film after incorporating LPP. The original hydrogen bonds between CMC chains that stabilized the film matrix were probably replaced with new hydrogen bonds between CMC molecules and LPP. Moreover, the hydrophobic character of LPP can decrease the amount of water absorbed during the preparation of CMC film loaded with LPP. It is known that water is a powerful plasticizer; thus, a decrease in water content can cause an increase in the brittleness of CMC film with LPP. In addition, a significantly lower E value for CMC-LPP material (E = 0.0078 MPa) suggests a higher deformation in this film under tensile or compressive stress than for the control CMC film (0.053 MPa). Therefore, adding LPP to the system causes an effect similar to a plasticizer, i.e., loosening the CMC polymeric net and decreasing intermolecular interactions responsible for film stiffness. The results of mechanical parameters indicate that CMC-LPP material had better mechanical properties for application in the packaging due to its higher elasticity (the lower E value). On the other hand, the LPP presence in CMC-based film can damage its structure and impair the film’s flexibility, resulting in decreased strain at break (ε). For comparison, the incorporation of free curcumin and curcumin-loaded nanohydrogels into CMC-based films led to a decrease in tensile strength (TS) values of CMC films (TS = 16.46, 11.23, and 9.87 MPa for CMC-based film, after the addition of free curcumin and curcumin-loaded nanohydrogels, respectively) [58]. Moreover, the presence of grapevine lignin extracted by deep eutectic solvents (DES) within the CMC-based films decreased the elongation at break from 4.67% to 3.13%, impairing the film’s flexibility and elasticity [56]. The obtained results indicate that the highest oxygen permeability was obtained for CMC-LPP samples with values in the range of 31,635.45–63,293.18 cm^3^/(m^2^·24 h) and 1503.59–2521.48 cm^3^/(m^2^·24 h) for CMC samples.

### 3.2. Antioxidant Properties of Films

Two QUENCHER (QUick, Easy, New, CHEap and Reproducible) methodologies involving forced solubilization of bound antioxidants capable of scavenging DPPH radical (QUENCHER_DPPH_) and reduction in copper (II)–neocuproine reagent (QUENCHER_CUPRAC_) without a preliminary time-consuming extraction process were applied for direct spectrophotometric determination of the antioxidant activity of the prepared CMC films with or without incorporated LPP. The advantage of the proposed QUENCHER procedure is the fact that, regardless of the hydrophobicity of antioxidants, surface reactions occur at the solid–liquid interface between antioxidant groups bound to insoluble matter (CMP films) and DPPH radical or chromogenic CUPRAC reagent.

As presented in Table 3, the results of antioxidant properties of the prepared CMC films determined by two modified analytical procedures, QUENCHER_DPPH_ and QUENCHER_CUPRAC_, differ significantly. The CMC film without LPP exhibited a low antioxidant activity of 2.26 and 0.92 μmol TE/g, determined by QUENCHER_DPPH_ and QUENCHER_CUPRAC_ assays, respectively. This suggests that CMC had a higher scavenging ability against DPPH radical than the reducing capacity of cupric to cuprous ions and formation of coloured copper (I)-neocuproine chelate. This variability can be explained by the hydrophobic character of CMC, making a control film before the incorporation of LPP better able to interact with DPPH radical dissolved in organic media, and thus, it has a higher affinity toward hydrophobic than hydrophilic antioxidants. Interestingly, hydroxyl groups in a molecular skeleton of CMC had the ability to eliminate DPPH free radicals and acted as hydrogen and electron donors in the obtained films. Similarly, the blank CMC films prepared by other authors revealed low and moderate scavenging activity analyzed by DPPH and 2,2′-azino-bis (3-ethylbenzothiazoline-6-sulphonic acid (ABTS) assays [14,56,59]. In contrast, Choi et al. [44] and Vidal et al. [45] reported that the control CMC films before fortification of natural extracts did not show an ability to scavenge DPPH radical. It is noteworthy that the LPP addition to the CMC film significantly increased its antioxidant properties (Table 3). The QUENCHER_DPPH_ and QENCHER_CUPRAC_ results for CMC films loaded with LPP were approximately 5 and 18 times higher than those for control CMC films. An explanation lays in LPP composition, i.e., the presence of various phenolic compounds such as gallic acid, protocatechuic acid, procyanidine B1, 3,4-dihydroxybenzaldehyde, 4-hydroxybenzaldehyde, catechin, and apigenin, which can trap free radicals by providing phenol hydrogen and/or via double bonds able to eliminate free radicals. Furthermore, these compounds could exert higher reducing power toward Cu (II)-neocuproine [41]. Additionally, a higher QUENCHER_CUPRAC_ result for CMC-LPP film than the QUENCHER_DPPH_ value for this enriched material confirm that the QUENCHER_CUPRAC_ assay is suitable for assessing the antioxidant potential of both hydrophilic and lipophilic antioxidants in the same film incorporating LPP, whereas the QUENCHER_DPPH_ assay uses a radical dissolved in organic media and thus has higher affinity toward hydrophobic than hydrophilic antioxidants in this prepared material.

On the other hand, the higher antioxidant properties of CMC film loaded with LPP indicate possible synergistic antioxidant interactions among CMC matrix film chains and antioxidant components present in the added LPP. Antioxidants can synergistically enhance the binding affinity of the enriched film components or weaken their interactions, affecting their antioxidant activity [60]. The enhancement of antioxidant features of fortified material depends on the delocalisation of electrons within the polyphenol benzene ring structure. The interactions with the matrix induce the ionization of phenolic hydroxyl groups, thereby enhancing the hydrogen supply capacity of the film system and improving the antioxidant properties of the bipolymer incorporating active agents. Additionally, the improved antioxidant features of polysaccharide–polyphenol composites can be explained by their ability to scavenge free radicals and chelate iron ions [60]. For comparison, other authors observed that the antioxidant activity of CMC films was rapidly enhanced after adding different natural extracts (apple skin and onion peel extracts and powders, extracts from green coffee oil by-products, grapevine lignins, and chickpea hull polysaccharides) rich in bioactive compounds with antioxidant properties [14,56,57,59,61].

### 3.3. Antibacterial Properties

**Figure 2:**
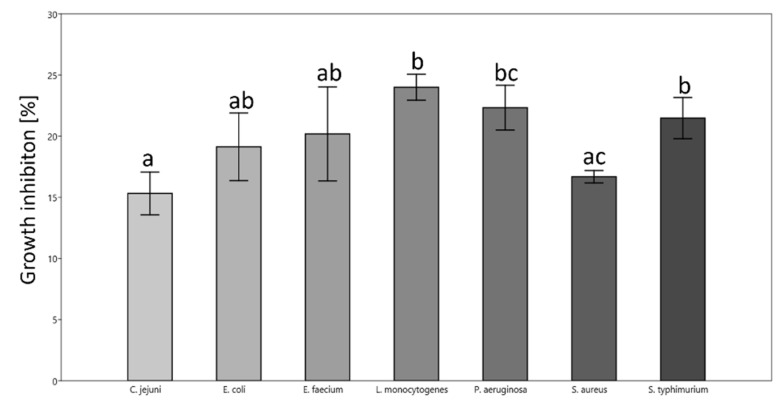
Growth inhibition of pathogenic bacteria in the presence of CMC-LPP film calculated based on the zone of inhibition of microorganism growth on solid medium LB. Different letters indicate significant statistical difference ± SD. One-way ANOVA with Tukey’s post hoc (*p* < 0.05; n = 3).

The tested bacteria showed differential sensitivity towards CMC-LPP films (Figure 2). CMC-LPP was most effective in inhibiting the growth of *L. monocytogenes* and least effective towards *C. jejuni* and *S. aureus*. The sensitivity of the tested bacteria can be arranged as follows (descending): *L. monocytogenes *> *S. typhimurium* > *P. aeruginosa* > *E. faecium* > *E. coli* > *S. aureus* > *C. jejuni*.

The drying process during the production of the tested CMC-LPP could possibly alter the concentration of some of the volatile organic compounds (VOCs), including phenolic compounds. As shown by Richert et al. [17], higher concentration of the birch tar added to the polymer results in more potent antibacterial activity due to the increased amount of phenol, betulin, cresol, chrysene, sterols, triterpenoid esters, triterpenoids, and acyl lipids. As mentioned above and described in more detail by Regert et al. [62], wood species used for pyrolysis significantly impacts the properties of the obtained products. Cedar tar severely inhibited the growth of *E. coli* and *Staphylococcus haemolyticus* [63]. The authors showed that after 2 h of incubation in liquid cultures, adding 5% cedar tar resulted in significantly reduced optical density and viable cell counts. Kizil et al. [64] demonstrated that crude extract of pine tar at a concentration of 80 µg/ml effectively inhibited the growth of *S. aureus*, *Streptococcus pyogenes*, *E. coli*, and the yeast *Candida albicans*. This information can be used for formulating membranes for specific purposes, i.e., a higher concentration of LPP for plastic used for wrapping computers and medical equipment in hospitals and operational rooms and smaller dosages for food packaging purposes. Due to their effectiveness at low concentrations, wood pyrolysis products can serve as a versatile antimicrobial substance, reducing the costs of developing and manufacturing new polymers. The addition of wood tar to polymers also increases their biodegradability, which may reduce the plastic pollution of the environment [21,39].

### 3.4. Cytotoxicity Analysis of Films

**Figure 3:**
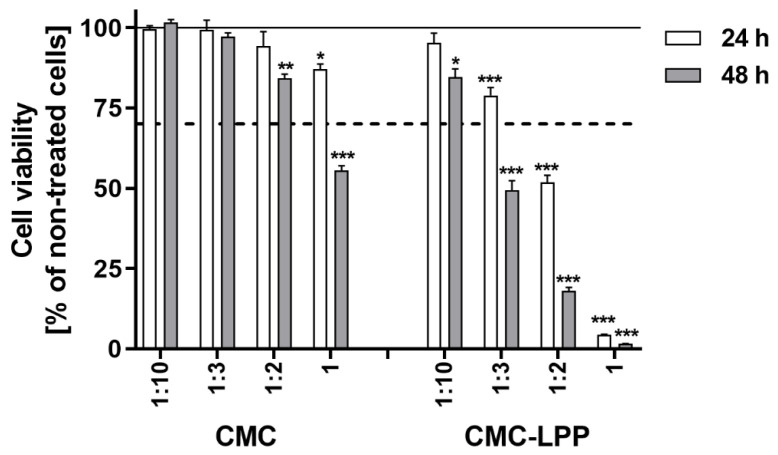
Assessment of L929 murine fibroblast survival following treatment with extracts prepared from CMC and CMC-LPP films. Cells were exposed to the extracts for 24 and 48 h, using four different dilution ratios: 1:10, 1:3, 1:2, and undiluted (1:1). Results are shown as the percentage of viable cells (mean ± SEM) relative to untreated controls. Statistically significant differences from the control group (considered 100%; indicated by a solid line) are marked with asterisks (*** *p* < 0.001, ** *p* < 0.01, * *p* < 0.05). A dashed line represents the cytotoxicity limit, defined as 70% cell viability under ISO 10993-5 guidelines.

The cytotoxic effect of the extracts derived from the tested films was evaluated using murine fibroblast cell line L929, which is one of the several well-suited cell lines recommended by the ISO 10993-5 [65] for the evaluation of the cytotoxicity of tested materials using the MTT assay (Annex C in ISO 10993-5). The results showed that the extracts derived from CMC films and CMC-LPP films significantly reduced the viability of L929 cells in a time-dependent and dilution-dependent manner (Figure 3). However, CMC samples induced a much less toxic effect on the L929 cells since cell viability was decreased only for the extract diluted by a ratio of 1:2 after 48 h (*p* < 0.01) and the undiluted extract both after 24 and 48 h (*p* < 0.05 and *p* < 0.001, respectively) in comparison with control cells. Moreover, the survival rate of cells was reduced to below 70% only for the cells stimulated with an undiluted extract derived from the CMC sample for 48 h (*p* < 0.001). In contrast, the cytotoxic effect for the CMC-LPP sample manifested by a reduction in cell viability to below 70% was observed for the extract diluted by a ratio of 1:3 after 48 h (*p* < 0.001), and this effect deepened with the increased concentration of the extract (Figure 3).

In the present study, the cytotoxicity of CMC and CMC-LPP samples was evaluated using an extract method that allows the assessment of the cytotoxicity of any leachable products from the tested materials. For extraction, the samples were placed in an incubator at 37 °C for 8 h in RPMI-1640 medium, which is in line with the ISO 10993-12 standard. For medical devices which are in short-term contact with skin or mucosa, and which are not implanted, this ISO norm allows an extraction time of less than 24 h, but not less than 4 h (ISO 10993-12 standard). The results from the MTT assay revealed that CMC-LPP samples induced a much greater cytotoxic effect on L929 cells than CMC samples. The findings of other authors showed that CMC was not cytotoxic up to high concentrations, which was observed towards human epithelial cell line Caco-2, hepatoma HepG2 cells [66] or human corneal epithelial cells (HCECs) [67]. Importantly, the differences in the evaluation of the cytotoxic concentration of CMC are consequences not only of the type of cell line used in experiments but also of the type of sample preparation procedure, the method used for cytotoxicity assessment, and the time of cell stimulation. Our results confirm these findings, showing that only the undiluted extract, and only after 48 h of stimulation, reduced cell viability to below 70%. In contrast, the extract derived from CMC-LPP samples showed cytotoxic activity towards L929 cells already for the extract diluted 1:3 after 48 h.

It is well established that the same components contributing to cytotoxicity are also responsible for the antibacterial and antioxidant properties of the film. This often represents a trade-off: the greater the biological activity, the higher the risk of some degree of cytotoxicity. The cytotoxicity of LPP from different wood was observed, among others, for Chinese hamster ovary (CHO) cells [59], human skin fibroblast cell line (HSF 1184) [68], embryonic fibroblast cell line NIH3T3 [69], or Jurkat T cells [70]. LPPs contain a wide range of cytotoxic compounds, including phenols, alcohols (e.g., methanol and ethanol), aliphatic hydrocarbons (e.g., hexane and heptane), polycyclic aromatic hydrocarbons, aldehydes, acetic acid, and other organic substances. Many of these components are known to exhibit significant cytotoxic effects [69,70,71]. In our previous studies, we specifically identified the presence of several of these component classes in the tested LPP samples, including phenolic compounds, aliphatic alcohols, ketones, aldehydes, and aliphatic hydrocarbons [34,45]. Each of these compound classes exhibits distinct, yet sometimes overlapping, mechanisms of toxicity. However, they generally exert their harmful effects through pathways such as cell membrane damage, oxidative stress induction, mitochondrial dysfunction, and the formation of DNA or protein adducts [72].

Our results showed that the carboxymethylcellulose matrix containing liquid pyrolysis products can be classified as a material for packaging applications. However, the pyrolysis products should be implemented in larger dilutions to plasticised CMC. Moreover, for materials intended for direct or indirect contact with food, it is essential to comply not only with general ISO cytotoxic standards but also with specific regulatory frameworks, such as those established by the European Food Safety Authority (EFSA; EU Regulation No. 10/2011) and the U.S. Food and Drug Administration (FDA 21 CFR 175–189). In accordance with these regulations, additional evaluations should be conducted, including migration testing to assess the potential transfer of substances into food products, and a comprehensive analysis of the chemical composition of the tested materials.

### 3.5. Biodegradability of Obtained Films

**Figure 4:**
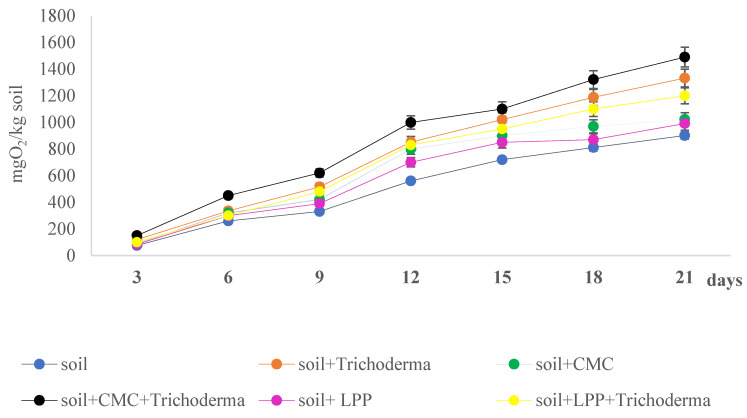
Biochemical oxygen demand (BOD) over 21 days in soil samples containing CMC or CMC-LPP films, with or without *T. viride* spores (n = 3 ± SE).

Fungi of the *Trichoderma* genus are widely distributed in various environments, especially in soil, where they interact with plants to promote their growth and participate in the biodegradation of crop residues [73,74,75]. Moreover, in previous studies, we have shown that *T. viride* participates in the acceleration of biodegradation of polycaprolactone, polyethylene terephthalate, and polylactide [76,77], hence the selection of the *T. viride* strain for this study. Oxygen consumption by microorganisms in each sample was proportional to the incubation time. The highest oxygen consumption throughout the experiment was noted for samples containing CMC film and *T. viride*, samples without films with *T. viride*, and CMC-LPP with *T. viride*, respectively (Figure 4). This indicates that CMC and CMC-LPP membranes are highly biodegradable, serving as sources of carbon for microbes present in the soil. This conclusion is further fortified with results from samples containing CMC and CMC-LPP without the addition of *Trichoderma* spores, i.e., the biological oxygen consumption in those samples was higher than in the control sample (soil). *Trichoderma* spp. shows great potential for biodegradation of various polymers with additional naturally derived substances. *Trichoderma atroviride* TN1 and *Trichoderma citrinoviride* TN3 metabolized phenolic acid-modified chitosan films in compost most effectively in comparison to native microbes present in the compost. These fungal strains exhibited high enzymatic activity (i.e., lipase, aminopeptidase, chitinase, β-1,3-glucanases) [31]. Similar results were obtained for polylactide (PLA) embedded with birch tar, where microbial metabolic activity and oxygen consumption were notably higher in compost samples when compared to control polymers (i.e., polylactide without addition of tar) [78]. Efficiency of the microbial biodegradation of polymers is differential between native microbes. BOD analysis of aliphatic polyesters, i.e., PLA and poly (ε-caprolactone) (PCL), embedded with birch bark tar showed lower oxygen consumption of native microbes in river water and soil when compared to PLA and PCL without the addition of tar. Moreover, the higher the concentration of tar in the polymer, the bigger the drop in oxygen consumption, suggesting a negative impact on the microbial activity and thus the lowered biodegradability of the produced polymers [39]. These results give a promising direction for new eco-friendly packaging materials, although there is an urgent need for further research in this area before these products can be placed on the market.

## 4. Conclusions

According to recent market analyses, the global biodegradable packaging market is expected to grow rapidly, driven by environmental regulations and consumer demand for sustainable materials, with CMC being a key component among polysaccharide-based films. However, challenges such as poor water resistance, mechanical fragility, and limited scalability remain major bottlenecks for large-scale application. To address these limitations, current research focuses on the chemical modification of CMC, composite formulations, and incorporation of bioactive agents that improve performance and functionality. The present study contributes to this development by exploring the integration of LPP—a phenolic-rich pyrolysis product—into CMC films to enhance bioactivity and degradability. The introduction of natural bioactive pyrolysis products to CMC-based materials resulted in the modification of their mechanical and physical properties. It was shown that the new material has high antioxidant and antibacterial properties, which is of great importance due to the need to limit the spread of pathogenic bacteria in the food and fodder industry, as well as to hinder the spread of infectious agents in healthcare systems. Furthermore, CMC-LPP films were shown to be biodegradable and can act as a source of additional carbon for soil microbes. The synergistic interaction of various LPP components acting as antioxidants and antimicrobial agents may prevent food spoilage caused by oxidation and microbial activity. Such an approach in the development of novel packaging materials may positively influence consumer acceptance of packaged food products, potentially extending their shelf life. Possibilities of LPP use in agriculture, forestry, medicine, cosmetology or veterinary medicine should be further explored to assess its safety and properties.

## Figures and Tables

**Table 1:** Abundance of microorganisms in the soil used for biochemical oxygen demand analysis.

Parameter	Abundance [CFU/g]
Heterotrophic bacteria	55 × 10^5^
Actinomycetes	42 × 10^4^
Fungi	33 × 10^3^

**Table 2:** Uniaxial tensile test results for CMC (control) and CMC-LPP films.

Film Sample	E ± SD * (MPa)	σ_max_ ± SD * (MPa)	ε ± SD * (%)
CMC	0.053 ± 0.017 ^b^	0.136 ± 0.016 ^b^	621.38 ± 58.28 ^b^
CMC-LPP	0.0078 ± 0.0016 ^a^	0.026 ± 0.004 ^a^	372.78 ± 41.85 ^a^

* n = 3; SD—standard deviation; different letters (a,b) within the same column indicate significant differences between mechanical properties of the studied films (one-way ANOVA and Tukey post hoc test, *p* < 0.05).

**Table 3:** Antioxidant activity of CMC films without and with liquid pyrolysis products (LPPs).

Film Sample	QUENCHER_DPPH_ ± SD * [μmol TE/g]	QUENCHER_CUPRAC_ ± SD * [μmol TE/g]
CMC	2.26 ± 0.19 ^a^	0.92 ± 0.04 ^a^
CMC + LPP	10.48 ± 0.39 ^b^	16.82 ± 0.73 ^b^

* n = 3; SD—standard deviation; different letters (a,b) within the same column indicate significant differences between antioxidant capacity of the studied CMC films determined by two analytical methods (one-way ANOVA and Tuckey post hoc test, *p* < 0.05).

## Data Availability

Relevant data applicable to this research are included in the paper and are also available upon request from the corresponding author.
